# Meeting Review: ESF Workshop on ‘Impact of Nucleic Acid Chemistry on Gene Function Analysis: Antisense, Aptamers, Ribozymes and RNAi’

**DOI:** 10.1002/cfg.186

**Published:** 2002-10

**Authors:** Kathrin Heermeier, Hans Prydz, Jutta Reinhard-Rupp, Joachim Engels

**Affiliations:** 1 Aventis Pharma Frankfurt Germany; 2 Biotechnology Centre of Oslo Oslo Norway; 3 University of Frankfurt Institute for Organic Chemistry Frankfurt Germany; 4 Aventis Pharma Industriepark Hoechst Bldg. G 879, 225 Frankfurt D-65926 Germany


**Comparative and Functional Genomics**
*Comp Funct Genom* 2002; **3**: 441–446.
Published online 29 August 2002 in Wiley InterScience (www.interscience.wiley.com). **DOI:** 10.1002/cfg.186

**Feature**

### Meeting Review: ESF Workshop on 'Impact of Nucleic Acid Chemistry on Gene Function Analysis: Antisense, Aptamers, Ribozymes and RNAi'

Hotel Rheinfels, St. Goar, Germany, 4–6 April 2002

Kathrin Heermeier,[1]* Hans Prydz,[2] Jutta Reinhard-Rupp[1] and Joachim Engels[3]
[1] *Aventis Pharma, Frankfurt, Germany*
[2] *Biotechnology Centre of Oslo, Oslo, Norway*
[3] *University of Frankfurt, Institute for Organic Chemistry, Frankfurt, Germany*

**Correspondence to:*
*Kathrin Heermeier, Aventis Pharma, Industriepark Hoechst, Bldg. G 879, 225, D-65926 Frankfurt, Germany.*
*E-mail: kathrin.heermeier@aventis.com*

Received: 24 May 2002
Accepted: 10 June 2002

### Introduction

The shortage of functional information compared to the abundance of sequence information characterizes today's situation in functional genomics. For many years the knock-down of a gene's product has been the most powerful way of analysing its function. In addition to the complete knock-out by homologous recombination, several different techniques have been developed to temporarily knock down gene expression through methods based on specific sequence recognition, such as knock-down by antisense oligonucleotides, ribozymes, aptamers or RNAi.

The ESF workshop on 'Impact of Nucleic Acid Chemistry on Gene Function Analysis' brought together researchers who use techniques that are different but highly related. It offered an opportunity for an in-depth discussion of recent progress and common problems. Antisense oligonucleotides, aptamers and ribozymes are techniques that have been used successfully for many years to validate targets. However, recent developments, such as increased tightness of binding (e.g. locked nucleic acids) or the combination of different methods (e.g. using aptamers to design ribozymes), have continued to improve the existing techniques. RNA interference (RNAi) is a defence mechanism of the cell against viruses. Since the exact mechanism of action within the cell is still unclear, RNAi was a particularly exciting topic at the workshop and was addressed in the largest number of presentations. Predictability of positional effects (accessibility of RNA) is a problem shared by all techniques using sequence-specific recognition and was the subject of quite controversial debates.

The meeting comprised over 50 people from 14 countries (13 European countries and the USA).

Copyright © 2002 John Wiley & Sons, Ltd.

### Session on antisense approaches and accessibility of RNA

**Susan M. Freier (Isis Pharmaceuticals, Carlsbad, USA)** gave an overview of the three different mechanisms of antisense oligonucleotides: RNase H mechanism, occupancy only, and non-RNase H cleavage. During her presentation, many parameters were discussed with respect to optimal site selection for antisense oligonucleotides. Oligos targeted to some feature types (UTR, CDS, intron) are active, but correlations still remain difficult, e.g. binding (thermodynamic processes) at a single site correlates with antisense activity. It could be demonstrated that calculated duplex free energy and sequence motifs correlate with the message inhibition. For antisense oligo design, additional aspects have to be considered, e.g. target specificity (are all isoforms known?), other species and certain motifs that support a non-antisense effect (e.g. G-quartets) [11]. In summary, it was stated that no single parameter demonstrated high correlation with cellular antisense activity, but each parameter could offer an improvement on the 'hit rate' and combinations of parameters could significantly improve hit rates.

Non-RNase H mechanisms: oligos selected for non-RNase H mechanisms could re-direct splicing to produce a variant mRNA and could redirect polyadenylation [15] when targeting polyadenylation signals. In addition, oligos targeting the 5′ cap could inhibit translation and RNA 'gap-mers' could be used to cleave target mRNA.

**Michael Gait (Medical Research Council, Cambridge, UK)** presented HIV-1 TAR (transactivation responsive element) as a target for inhibition by antisense oligos. Efficient inhibition of TAR RNA in HeLa cell nuclear extracts was proven for oligos such as 2′-*O*-methyl-oligoribonucleotides (OMe), chimeric OMe/LNA oligos and PNA. TAR binding strength was shown to be dependent on buffer conditions, and oligo concentrations of 100–200 nM resulted in 50% inhibition of transcription. The attachment of FAM was introduced as a new general procedure for synthesis of 3′-labelled oligonucleotides. For delivery into cells, the group tested different delivery systems in HeLa cells stably transfected with three plasmids (TAT gene, firefly luciferase and *Renilla* luciferase under three different promoters) [1]. Also of interest is a new class of cationic gemini surfactants, including a new compound from GlaxoSmithKline (GS 11) [12] which has proved to be very efficient. In the last part of the presentation, Mike Gait introduced a delivery system using peptide conjugates for cell translocation by a non-endosomal route. The strategy for conjugation was proposed as a total stepwise solid-phase synthesis of peptide–oligo conjugates.

**Georg Szakiel (Institute for Molecular Medicine, Lübeck, Germany)** described a new concept of computational antisense oligo design based on a systematic analysis of secondary structure predictions of target RNA. For improved cellular delivery, a hybrid approach of additional nucleotide sequence elements combined with an indicator gene showed the feasibility of this system.

**Judith van Deutekom (Leiden University Medical Centre, The Netherlands)** presented work on a sequence-based exon-skip therapy for Duchenne muscular dystrophy (DMD). Dystrophin frame-shift mutations cause different forms of mild (Becker muscular dystrophy, BMD) or severe (DMD) muscle degeneration. Antisense oligos were used to correct these back into frame by inducing exon skipping. This led to normal amounts of properly localized dystrophin in a mouse model with DMD patient-derived muscle cells. The future could be a gene correction therapy using antisense oligos for exon skipping.

**Stefan Amberg (University of Frankfurt, Germany)** discussed the inhibition of hepatitis C viral gene expression by differently modified antisense oligos. The oligodeoxynucleotides (ODNs) had polar (phosphorothioate) or non-polar (benzyl-, 2-phenylethyl-, 4-phenylbutylphosphonate) modifications and were synthesized via phosphoramidite chemistry. The best inhibitory effect *in vitro* and in cell culture (HepG2) was demonstrated for terminally modified benzyl-oligodeoxynucleotides.

**Gunther Hartmann (University of Munich, Germany)** addressed the modulation of malignant B cell activation and bcl-2-mediated apoptosis by antisense ODN and immunostimulatory CpG ODN (ODN containing unmethylated CG-dinucleotides). In the mouse tumour model, it was demonstrated that CpG ODN has potential as a therapeutic due to its immunostimulatory properties.

**Bertrand Tavitian (INSERM Orsay, France)** finished the first session by demonstrating a different use of oligos, namely by adapting oligos as radiotracers for positron emission tomography

Copyright © 2002 John Wiley & Sons, Ltd.

(PET). The sensitivity of PET combined with radiolabelled oligos to address gene expression resulted in an impressive application in animal tumour models. Several video clips demonstrated the potential of this imaging technology.

### Session on RNAi in plants and model organisms

**Scott M. Hammond (Cold Spring Harbor Laboratory, New York, USA)** briefly described the history of RNA interference, with emphasis on the founder articles by Fire *et al.* [5] and on the discovery of short interfering RNA (siRNA) by Hamilton and Baulcombe [6], before going on to describe the biochemical characterization work on RNAi components done by Gregory Hannon's group [7, 2, 8]. In short, defining the RNase-III-like nuclease Dicer, defining the mRNA degrading complex RISC, and finally, isolating enough of the RISC complex to allow microsequencing of components, thereby finding the PAZ/PIWI protein Ago-2. Unpublished work described included the microsequencing of another RISC component, Vig, which has no known domains, but which has a human homologue that is known to bind mRNA, and one component, CG7008, that is a potential candidate for the actual mRNA cleaving protein, termed SLICER. Future applications described by Hammond included: (a) a cost-effective T7-based expression system for production of siRNA from short DNA templates; (b) a plasmid construct, pSHAG, for expressing short hairpins that are processed to siRNA; and (c) an informatic/high-throughput scheme for screening large numbers of genes (selected sets, e.g. in cancer, apoptosis, lethality, drug research) *in vivo* by transfecting in T7 transcribed siRNA in 96-well plates.

**Marcel Tijsterman (Netherlands Institute for Developmental Biology, Utrecht, The Netherlands)** related the discovery in Ronald Plasterk's group of the linkage between mutator *C. elegans* strains containing activated transposons and RNA silencing [10, 13, 14]. The isolated mutants were shown to be homologues of genes involved in RNAi in other organisms, notably rde-1, rde-4 and mut-7. Turning the focus to RNA interference in *C. elegans*, more mutants and/or homologues of known RNAi genes were investigated, uncovering the basic mechanisms of siRNA production *in vivo* and sterility of Dcr-mutants, and mapping pathways of genes needed for siRNA production. A remarkable mechanism producing new siRNA by extending 21–40 nt antisense RNAs on an mRNA template was discovered, termed 'transitive RNA' or 'secondary siRNA'. The RdRP-homologues rrf-1, rrf-2 and rrf-3, seem to be involved, with rrf-1 being necessary and rrf-3 actually seeming to be an inhibitor of RNAi, making the rrf-3 strain interesting for enhanced RNA interference screens in *C. elegans*.

**Martin Tabler (Institute of Molecular Biology and Biotechnology, Heraklion, Greece)** covered three topics. In the first section the effects of various modifications on the activity of anti-notch siRNAs were reported [3]. The absence of 5′ phosphate lowered penetrance of the Notch phenotype but did not affect its expressivity. siRNAs were found to be tolerant for a single mutation, while a double-mutation significantly lowered both penetrance and expressivity. DNA was not tolerated in either strand of the siRNA. Tabler then proceeded to describe the expression of a 'pan-handle' dsRNA in transgenic *N. tabacum* plants. These transgenic plants displayed three different phenotypes, in which the degree of virus resistance correlated with the expression of transgene-derived siRNAs. Silencing was further found to be stable between $T_0$ and $T_1$ generations. Finally, the induction of RNAi in *C. elegans* embryos following injection of an extract prepared from silenced plants was reported [4]. Following size fractionation of the extract, the highest silencing activity was found in the 81–90 nt size range. The RNA responsible for this activity appeared to be present at very low concentration. Tabler concluded his presentation by hypothesizing that a hairpin RNA of 85 nt, resulting from aberrant transcription, might be responsible for systemic spreading of silencing.

In a short presentation, **Yuanhuai Han (University of Nottingham, UK)** reported inverted repeat (IR)-dependent silencing of an ACC oxidase 1 sense transgene in tomato plants [9]. Silencing was accompanied by the production of small antisense RNAs of 21–28 nt. These asRNAs were present at much higher concentrations with the sense than the antisense transgene. Furthermore, the asRNAs appeared to be produced preferentially from the 5′ end of the gene.

Copyright © 2002 John Wiley & Sons, Ltd. 

### Session on RNAi in mammalian cells and humans

**Tom Tuschl (Max Planck Institute for Biophysical Chemistry, Göttingen, Germany)** gave an overview of the extensive work his group has done on RNAi in mammalian cells. When using short, approximately 21 nt RNA instead of long RNA, no interferon response is triggered in the cells. Optimal results are obtained with 2 nt 3′ overhangs and starting with a G at the upstream end. He demonstrated that the cleavage of siRNA is exactly in the middle of the sequence and excluded the generation of secondary siRNA species. The second part of his talk focused on microRNA. MicroRNAs are excised from 30 bp stem–loop structures by a mechanism related to RNAi. They may function as negative regulators, guiding nucleases to homologous mRNA. Cloning and sequencing of endogenous RNAs led to the identification of about 70 novel microRNAs.

**Mohammed Amarzguioui (Biotechnology Centre of Oslo, Norway)** discussed the efficacy and duration of RNAi in mammalian cells in respect to several parameters. He observed strong positional effects that did not correlate with the predicted target site secondary structure or siRNA GC content. Mismatches were tolerated in siRNA, resulting in gradual attenuation in the rate and extent of depletion. The silencing effect in his experiments was transient, reaching its maximum after 24 h and recovering gradually after 72 h. He found no evidence for a propagation of RNAi (secondary siRNA) in human cells.

**Stefan Limmer (Ribopharma AG, Bayreuth, Germany)** presented *in vivo* gene silencing through RNAi. Transgenic mice expressing the green fluorescent protein (GFP) gene were repeatedly injected with siRNA. A substantial decrease of the GFP mRNA level was achieved in kidney, heart and serum. Several other organs (small intestine, pancreas, lung and stomach) showed varying degrees of inhibition, while brain and liver appeared to be hardly accessible.

**Kathrin Heermeier (Aventis Pharma, Frankfurt, Germany)** demonstrated downregulation of target genes using RNAi in a variety of different cells, both of insect and mammalian origin. The second part of her talk compared antisense and RNAi data obtained in human endothelial cells. Gene silencing of comparable levels was achieved by both methods. While she described the limitations of both methods as similar (need for cell transfection and short-lived target proteins), the success rate was higher using siRNA than antisense.

**M. Famulok (University of Bonn, Germany)** demonstrated the activity of intramers expressed by a transgenic virus using the inhibition of cytohesin 1, a guanine–nucleotide exchange factor, as an example. Successful aptamers lead to a reorganization of the actin cytoskeleton in T cells. Specifically binding aptamers can then be utilized to design allosteric ribozymes that specifically cleave the target sequence. These ribozymes can be used in high-throughput assays for finding small molecules that bind the target at the selected domain.

**Astrid E. Klöpffer (J. W. Goethe University, Frankfurt, Germany)** reported the synthesis and incorporation of fluoro-modified universal bases into a selected anti-HIV polymerase hammerhead ribozyme. The substitution with the modified nucleobases resulted only in a small reduction of the catalytic efficiency of the ribozyme. Comparable results were obtained with a ribozyme containing one mismatch base pair.

**Ettore Luzi (Polo Scientifico, Firenze, Italy)** used a library of *in vitro* selected hammerhead-like ribozymes with randomised arms to identify ribozyme-accessible sites on the HIV-1 LTR. A modified ribozyme with 2′ modifications and phosphorothioate groups was designed against such a site. The modified ribozyme showed an extended range of triplets that could be cleaved.

### Round table discussion

Friday evening was reserved for a round table discussion with no predetermined topic. RNAi, and how it compares to the other methods, was the major focus of the evening. For a while, the debate revolved around the presence or absence of RNA-dependent RNA-polymerase (RdRP) in various organisms that may or may not have a role in RNAi. There was consensus that RdRP is present in plants and in *C. elegans*; there is no definitive proof for RdRP activity in either *Drosophila* or mammals.

The pros and cons of antisense and RNAi in the experimental knock-down of targets was another controversial topic. All agreed that the

Copyright © 2002 John Wiley & Sons, Ltd. 

lack of certain design rules that would reliably result in effective oligonucleotides is a problem in both techniques.

Since the first results using RNAi *in vivo* are out, the round table speculated on the possibility of using RNAi in therapy. Potential side effects could occur: when the system is flooded with siRNA, the naturally occurring RNA degradation by microRNA might be compromised.

### Session on alternative approaches for gene silencing

The session started with **John J. Rossi (Beckman Research Institute of the City of Hope, CA, USA)**. His main interest is centred on gene therapy approaches directed towards HIV. Here he uses CD $34^{+}$ haematopoietic stem cells, which are transduced by tat or rev specific ribozyme carrying vectors. These constructs have been in clinical evaluation for 2 years. Since it is obvious now that Tat and Rev proteins have nuclear localization, the group developed Pol III expression systems where an anti-HIV ribozyme is in the context of U16 snoRNA. They also constructed vectors based on Pol III expression to deliver 21–23 nt RNAs into the nucleus. Here for the first time a 4-log reduction in p24 antigen production was observed for weeks. For therapeutic purposes, combinations of ribozymes and RNAi, for example, or even decoys, are a future line of research.

The advantages of structured RNAs for binding cellular targets (so-called 'aptamers') have been addressed by **Jean-Jacques Toulmé (University of Bordeaux, France)**. They selected aptamers against HIV-TAR and found a complementary 59 nt hairpin to the apical loop. The RNA aptamer, which had a G-A pair closing the loop, had an affinity ($K_d$) of 20 nM. When trying to stabilize the aptamer by chemical modification, the L-enantiomer failed, but the 3′-5′-phosporamidate was successful. It was not only comparably bioactive, but also nuclease-resistant. The above concept also holds true for silencing hepatitis C virus RNA. Here addressing the 3′-untranslated region, an aptamer with 70 nM $K_d$ could be obtained.

**Stefan Schulte-Merker (Artemis/Exelixis, Tübingen, Germany)** presented a functional genomics approach in a whole organism. Here zebrafish were used for reverse genetics. Injecting 25-mer morpholino-based antisense oligonucleotides (directed against the 5′ UTR) into zebrafish embryos resulted in up to 98% silencing. The effect is active after 10 h, and lasts up to 5 days. Their main effort is now directed towards studying angiogenesis, e.g. VEGF knockouts. Altogether, 4531 genomes were screened and 746 mutants selected; 150 genes have been isolated.

Following this, the latest developments in the chemical synthesis of antisense oligos were presented. Here the hexitol nucleic acids (HNA) and the locked nucleic acids (LNA) were discussed by **Piet Herdewijn (Medical Chemistry, Leuven, Belgium)** and **Jesper Wengel (Odense University, Denmark)**, respectively. Piet Herdewijn tested hexo-pyranose analogues and found that HNA was a powerful steric blocker for mRNA, and a poor substrate for RNase H. He also showed that HNA triphosphates are accepted by Vent-Polymerase.

Jesper Wengel fixed the ribose moiety of RNA in the A form, using a 2′-4′-bridge. Such structurally preformed nucleosides are very strong RNA-binders (conformationally or entropically driven). NMR structures have been shown to support their ideal conformation. Since they are not a substrate for RNase H, they are used as gap-mers to inhibit mRNA. Some cell experiments using the oestrogen receptor in MCF-7 cells convincingly showed their antisense potential. These LNA-analogues seem to be excellent candidates to be combined with other modifications in order to stabilize the oligo-RNA hybrids.

### Concluding remarks

This workshop brought together scientists involved in the synthesis of nucleic acids with scientists that use their results. Perhaps as a consequence discussions were particularly lively and fruitful. The workshop was small (approximately 50 participants) and the topic of antisense-based techniques rather specific. Since equal time was given to presentations and discussions, there was room for detailed discussions of experimental approaches and possible future improvements.

Originally we had planned to cover several different techniques with equal weight within the workshop. However, after the invited speakers

Copyright © 2002 John Wiley & Sons, Ltd.

had sent in the titles of their talks it turned out that RNAi was the centre of attention for many researchers. Therefore, two of the four sessions were devoted to this technique. RNAi represents the latest development in the field of downregulation of gene expression through sequence-specific recognition. The recent shift of interest towards RNAi illustrates both the speed with which the field changes, and how difficult it is to plan for the future. If a similar workshop is to be organized 2 years from now, what should the focus be? The participants of the workshop were unable to predict this. While technologies change, there will certainly be interest in studies of gene function for some time to come and we will continue to knock down genes one way or the other.

### Acknowledgements

The organisers would like to thank the ESF for financing the workshop and Aventis Pharma for generous sponsorship. Special thanks go to Dr Annette Martin who provided priceless advice and helped with the organization.

### References

1. Arzumanov A, Walsh AP, Rajwanshi VK, *et al*. 2001. Inhibition of HIV-1 Tat-dependent transactivation by steric block chimeric 2′-*O*-methyl/LNA oligoribonucleotides. *Biochemistry* **40**(48): 14 645–14 654.
2. Bernstein E, Caudy AA, Hammond SM, Hannon GJ. 2001. Role for a bidentate ribonuclease in the initiation step of RNA interference. *Nature* **409**(6818): 363–366.
3. Boutla A, Delidakis C, Livadaras I, Tsagris M, Tabler M. 2001. Short 5′-phosphorylated double-stranded RNAs induce RNA interference in *Drosophila*. *Curr Biol* **11**(22): 1776–1780.
4. Boutla A, Kalantidis K, Tavernarakis N, Tsagris M, Tabler M. 2002. Induction of RNA interference in *Caenorhabditis elegans* by RNAs derived from plants exhibiting post-transcriptional gene silencing. *Nucleic Acids Res* **30**(7): 1688–1694.
5. Fire A, Xu S, Montgomery MK, *et al*. 1998. Potent and specific genetic interference by double-stranded RNA in *Caenorhabditis elegans*. *Nature* **391**(6669): 806–811.
6. Hamilton AJ, Baulcombe DC. 1999. A species of small antisense RNA in posttranscriptional gene silencing in plants. *Science* **286**(5441): 950–952.
7. Hammond SM, Bernstein E, Beach D, Hannon GJ. 2000. An RNA-directed nuclease mediates post-transcriptional gene silencing in *Drosophila* cells. *Nature* **404**(6775): 293–296.
8. Hammond SM, Boettcher S, Caudy AA, Kobayashi R, Hannon GJ. 2001. Argonaute2, a link between genetic and biochemical analyses of RNAi. *Science* **293**(5532): 1146–1150.
9. Han Y, Grierson D. 2002. Relationship between small antisense RNAs and aberrant RNAs associated with sense transgene mediated gene silencing in tomato. *Plant J* **29**(4): 509–519.
10. Ketting RF, Haverkamp TH, van Luenen HG, Plasterk RH. 1999. Mut-7 of *C. elegans*, required for transposon silencing and RNA interference, is a homolog of Werner syndrome helicase and RNaseD. *Cell* **99**(2): 133–141.
11. Lesnik EA, Freier SM. 1995. Relative thermodynamic stability of DNA, RNA, and DNA:RNA hybrid duplexes: relationship with base composition and structure. *Biochemistry* **34**(34): 10 807–10 815.
12. McGregor C, Perrin C, Monck M, Camilleri P, Kirby AJ. 2001. Rational approaches to the design of cationic gemini surfactants for gene delivery. *J Am Chem Soc* **123**(26): 6215–6220.
13. Sijen T, Fleenor J, Simmer F, *et al*. 2001. On the role of RNA amplification in dsRNA-triggered gene silencing. *Cell* **107**(4): 465–476.
14. Tijsterman M, Ketting RF, Okihara KL, Sijen T, Plasterk RH. 2002. RNA helicase MUT-14-dependent gene silencing triggered in *C. elegans* by short antisense RNAs. *Science* **295**(5555): 694–697.
15. Vickers TA, Wyatt JR, Burckin T, Bennett CF, Freier SM. 2001. Fully modified 2′ MOE oligonucleotides redirect polyadenylation. *Nucleic Acids Res* **29**(6): 1293–1299.

Copyright © 2002 John Wiley & Sons, Ltd. 
